# Shikonin Attenuates Acetaminophen-Induced Hepatotoxicity by Upregulation of Nrf2 through Akt/GSK3β Signaling

**DOI:** 10.3390/molecules24010110

**Published:** 2018-12-29

**Authors:** Huachao Li, Yueming Chen, Jiahao Zhang, Xiangcui Chen, Zheng Li, Bing Liu, Luyong Zhang

**Affiliations:** 1 Department of Pharmacology, School of Pharmacy, Guangdong Pharmaceutical University, Guangzhou 510006, China; rehclee27@163.com (H.L.); yuemchen@163.com (Y.C.); avlsunny@126.com (J.Z.); 18766979213@163.com (X.C.); 2 Guangzhou Key Laboratory of Construction and Application of New Drug Screening Model Systems, Guangdong Pharmaceutical University, Guangzhou 510006, China; li.zheng.sky@163.com; 3 Key Laboratory of New Drug Discovery and Evaluation of Ordinary Universities of Guangdong Province, Guangdong Pharmaceutical University, Guangzhou 510006, China; 4 The Center for Drug Research and Development, Guangdong Pharmaceutical University, Guangzhou 510006, China

**Keywords:** shikonin, acetaminophen, Nrf2, PI3K/Akt pathway, GSK-3β, hepatotoxicity

## Abstract

Acetaminophen (APAP) overdose-induced acute liver damage is mostly due to overwhelmingly increased oxidative stress. Nuclear factor-erythroid 2-related factor2 (Nrf2) plays an important role in alleviating APAP hepatic toxicity. Shikonin (SHK) enhances Nrf2 in multiple lines of normal cells. Nevertheless, whether SHK protects against APAP-induced liver toxicity remains undefined. This study found SHK defended APAP-induced liver toxicity, as well as reversed the levels of serum alanine/aspartate aminotransferases (ALT/AST), liver myeloperoxidase (MPO) activity, and reactive oxygen species (ROS), while it enhanced the liver glutathione (GSH) level in APAP-treated mice. SHK rescued the cell viability and GSH depletion, but neutralized oxidative stress in APAP-treated human normal liver L-02 cells. Mechanically, SHK increased Nrf2 expression in the exposure of APAP at the protein level but not at the mRNA level. Inhibition of Nrf2 blocked the SHK effect in APAP-treated hepatocytes. Furthermore, SHK improved Nrf2 stability through stimulating PI3K/Akt pathway, thus inhibiting GSK-3β. In vivo studies confirmed the close correlation of liver protection of SHK against APAP and Akt/GSK-3β/Nrf2 pathway. In conclusion, this study reveals that SHK prevents APAP hepatotoxicity by upregulation of Nrf2 via PI3K/Akt/GSK-3β pathway. Therefore, SHK may be a promising candidate against APAP-induced liver injury.

## 1. Introduction

Acute liver failure caused by acetaminophen (APAP) overdose is the common and main reason for drug-induced liver injury (DILI) worldwide [1]. APAP is metabolized into a reactive metabolite N-acetyl-p-benzoquinone imine by the liver cytochrome P450, which depletes cellular glutathione (GSH), leading to oxidative stress and resultant liver damage [2,3]. Therefore, there is an urgent need for an effective oxidative stress-targeted therapeutic strategy against APAP-induced liver toxicity. 

Excessive reactive oxygen species (ROS) production facilitates a variety of oxidative stress responses, thus inducing cell death and advancing APAP hepatotoxicity [4]. Nuclear factor erythroid-2-related factor 2 (Nrf2), belonging to the cap′n′collar (CNC) family and/or CNC-bZIP proteins, maintains redox homeostasis through activating detoxifying enzymes by binding to antioxidant response elements (AREs) [5]. Activation of the Nrf2/ARE pathway plays an important role in alleviating liver toxicity caused by APAP [6]. Nrf2-deficient mice display highly susceptible to APAP hepatotoxicity [7]. Thus, Nrf2 may be a promising target for attenuating APAP-induced liver injury, by combating oxidative stress. 

Shikonin (SHK) is a natural product extracted from the roots of *Lithospermum erythrorhizon* [8]. Recent studies have shown that SHK can prevent septic acute kidney injury in mice induced by LPS via Nrf2 activation [9] and inhibit oxidized LDL-induced monocyte adhesion via up-regulation of Nrf2-mediated antioxidation in EA. hy926 endothelial cells [10]. Moreover, SHK has been confirmed to attenuate concanavalin A-caused acute liver injury in mice [11]. and protect lipopolysaccharide/D-galactosamine-induced acute liver injury [12]. However, the role of SHK in APAP-induced hepatic toxicity has not yet been defined. In the present study, we explored the potentially protective effects and the mechanism of SHK in APAP-induced hepatotoxicity in vitro and investigated the function of SHK against APAP-induced acute liver injury in vivo.

## 2. Results

### 2.1. Shikonin (SHK) Prevented Acetaminophen (APAP)-Induced Liver Injury In Vivo

**Figure 1:**
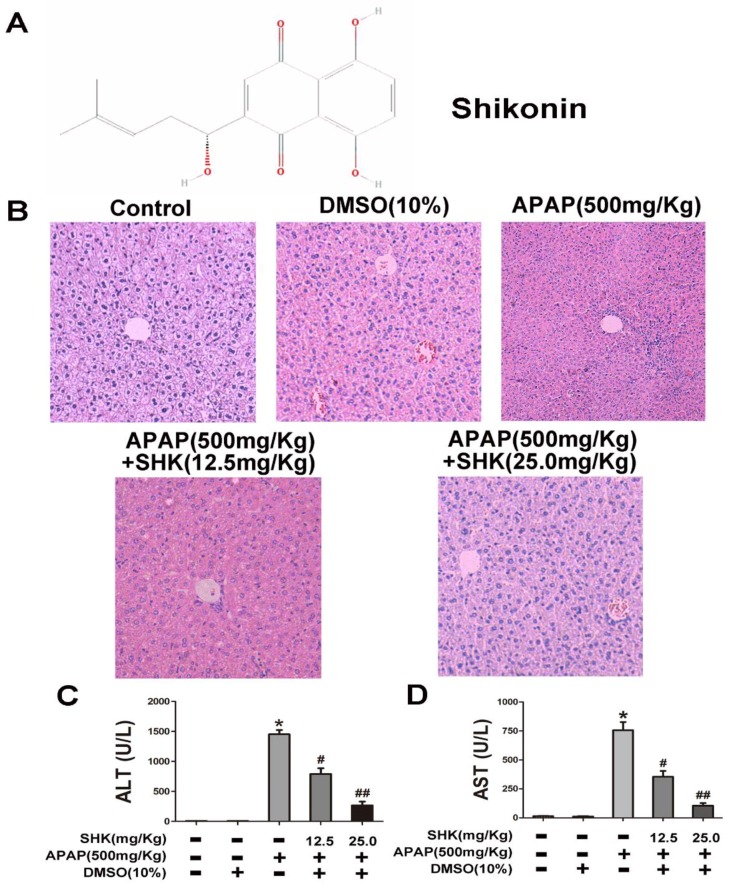
Effects of shikonin (SHK) on acetaminophen (APAP)-induced liver injury. (**A**) The chemical structure of SHK. (**B**) Representative histological images of hematoxylin and eosin (H&E) staining of liver tissues obtained in different groups. (**C**,**D**) Mice were administered SHK i.p. for 1 h, and then injected with APAP for 9 h. Then, the serum levels of serum alanine/aspartate aminotransferases (ALT and AST) were measured. All of the data shown represent the average from five independent experiments. * *p* < 0.05 versus the control group; # *p* < 0.05 and ## *p* < 0.01 versus the APAP group.

The structure of SHK with the molecular weight of 288.3 g/mol used in this study was shown in Figure 1A. We first examined the liver injury histopathologically to observe the protective effects of SHK. Figure 1B showed that mice treated with APAP exhibited severe liver injury, reflected by necrosis of hepatocytes and destruction of liver structure, and SHK (12.5 and 25.0 mg/kg) treatment antagonized these effects. As shown in Figure 1C,D, APAP increased the serum levels of serum aspartate/alanine aminotransferases (AST and ALT), which was significantly reversed by SHK. 

**Figure 2:**
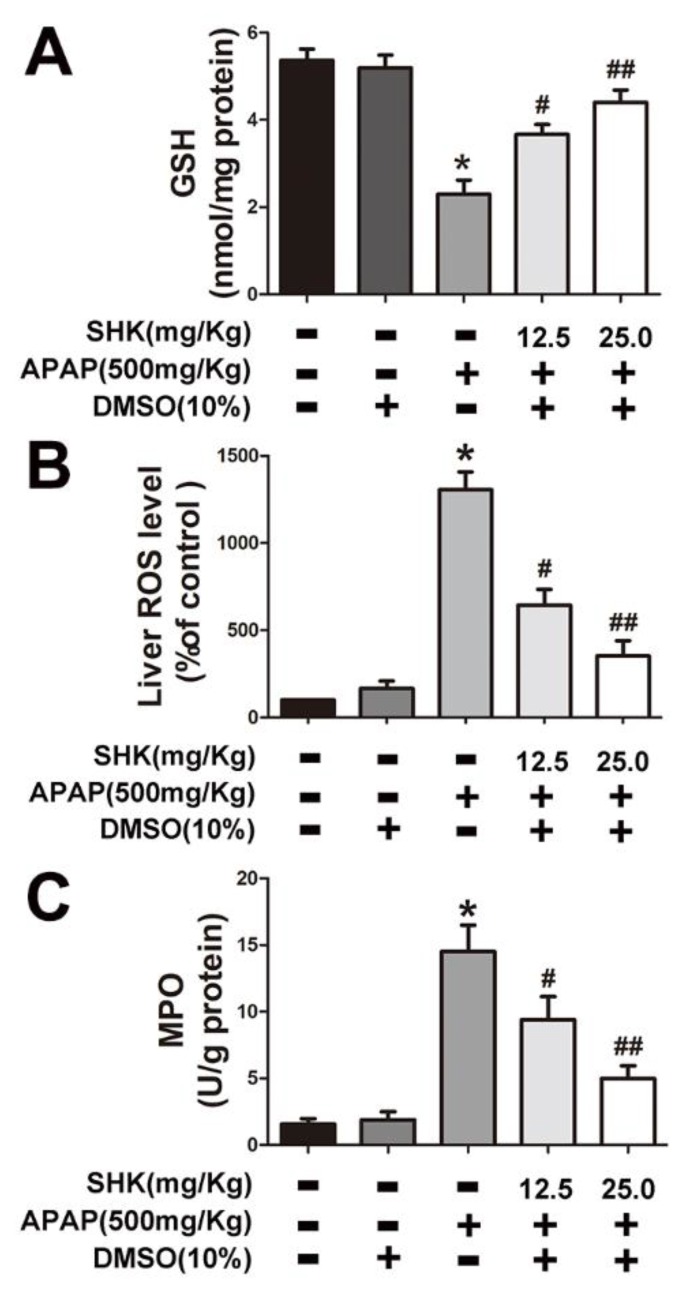
Effects of SHK treatment on APAP-induced glutathione (GSH), reactive oxygen species (ROS) and myeloperoxidase (MPO) production in mice. Mice were injected with SHK (12.5 and 25 mg/Kg) for 1 h, and then injected with APAP (500 mg/Kg) for 9 h. (**A**) The effect of SHK on liver GSH production in APAP-treated mice. (**B**) SHK reduced the accumulation of APAP-induced ROS in liver. (**C**) SHK inhibited APAP-induced MPO production in liver. All of the data shown represent the average from five independent experiments. * *p* < 0.05 versus the control group; # *p* < 0.05 and ## *p* < 0.01 versus the APAP group.

Next, we sought to determine the effect of SHK on APAP-induced oxidative stress in mice liver tissues. The results indicated that APAP treatment led to a significant decrease in GSH content, while increased ROS production and MPO activity (Figure 2A–C). However, these effects were ameliorated or reversed by SHK administration. 

Taken together, these results strongly support the idea that SHK effectively protects against APAP-induced oxidative stress and liver injury. 

### 2.2. SHK Inhibited APAP-Induced Hepatic Toxicity and Oxidative Stress in Hepatic L-02 Cells

**Figure 3:**
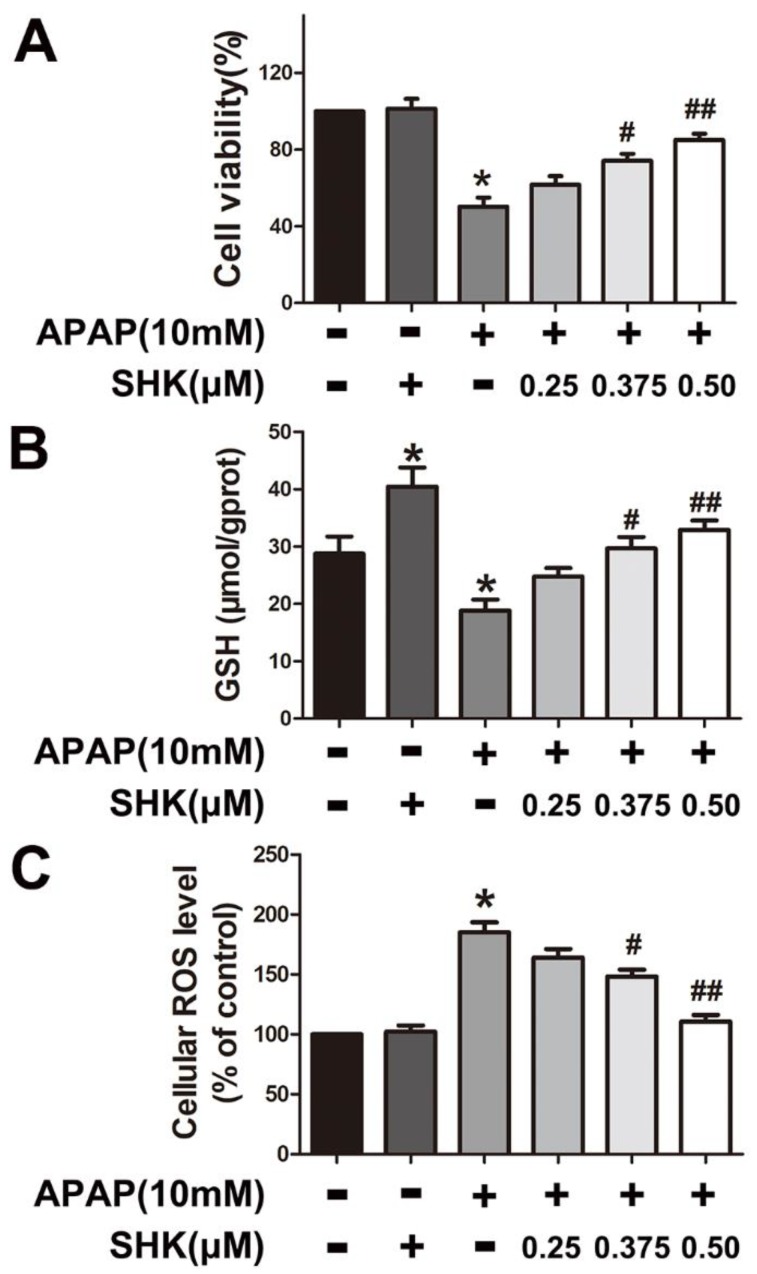
Effects of SHK on cell viability and oxidative stress in L-02 cells. (**A**) L-02 cells were treated with increasing concentrations of SHK (0.25, 0.375 and 0.50 μM) for 1 h, then use APAP (10 mM), cultured for 24 h, and then cell viability was evaluated by MTT assay. (**B**,**C**) L-02 cells were treated with SHK for 1 h, then treated with APAP (10 mM) and cultured for 8 h together, and then GSH and ROS was assessed using a commercial GSH test kit and 2houghdichlorofluorescin diacetate (DCF-DA) fluorescence assay, respectively. All of the data represent the average from three independent experiments. * *p* < 0.05 versus the control group; # *p* < 0.05 and ## *p* < 0.01 versus the APAP group.

To explore the potential inhibitory effect of SHK on APAP hepatic toxicity, the viability of hepatic L-02 cells after individual treatment after 24 h was measured by MTT assay. Figure 3A showed that APAP (10 mM) significantly decreased cell viability compared with control, which was inhibited by SHK (0.375 and 0.5 μM) pretreatment for 1 h. In addition, SHK (0.375 and 0.5 μM) pretreatment markedly rescued the GSH depletion caused by 8-h APAP treatment (Figure 3B). 

We further determined the effect of SHK against APAP-induced oxidative stress. The results showed that APAP enhanced cellular ROS level in L-02 cells after 8-h treatment, whereas SHK (0.375 and 0.5 μM) obviously reduced ROS accumulation induced by APAP (Figure 3C).

### 2.3. SHK-Reduced APAP Hepatic Toxicity is Dependent on Nrf2 in L-02 Cells

**Figure 4:**
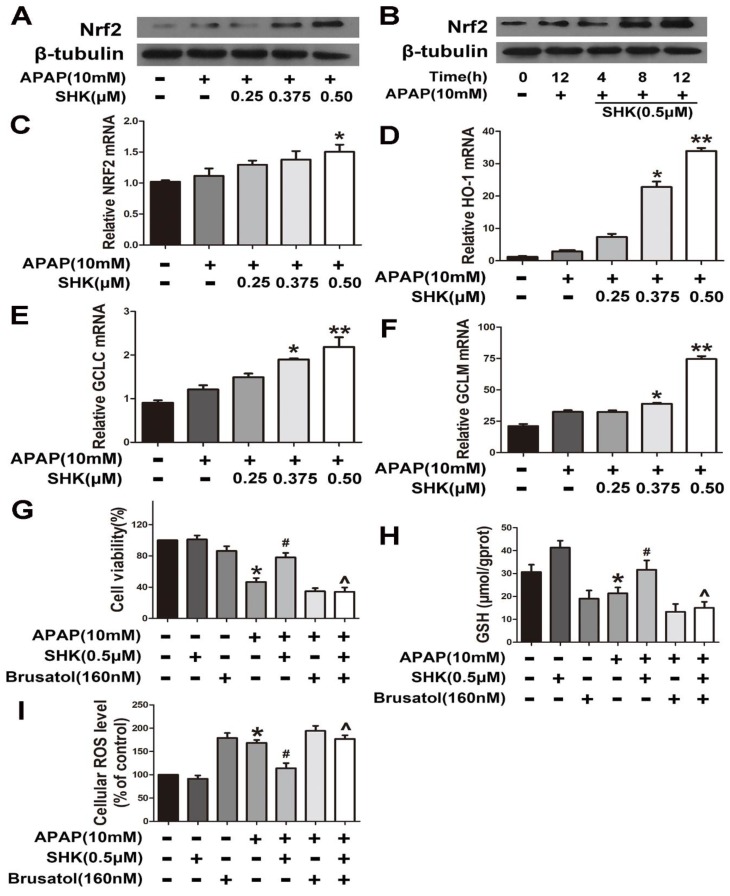
Effects of SHK are dependent upon Nrf2 in L-02 cells. (**A**,**B**) L-02 cells were treated with SHK for the indicated durations, and then the protein levels were examined by western blot analysis. (**C**–**F**) L-02 cells were treated with increasing concentrations of SHK for 1 h followed by 4-h APAP treatment, and then the mRNA expression of Nrf2, *HO-1*, *GCLc* and *GCLm* was detected by real-time polymerase chain reaction (PCR). (**G**–**I**) Cells were pretreated with brusatol (160 nM) for 2 h, followed by treatment with SHK for 1 h, and then the cells were exposed to APAP for 24 h. Cell viability, GSH and ROS level was assessed. All of the data shown represent the average from three independent experiments. * *p* < 0.05 versus the control group; # *p* < 0.05 versus the APAP group; ^ *p* < 0.05 versus the SHK plus APAP group.

As mentioned above, SHK as an Nrf2 activator controls oxidative stress in multiple types of normal cells. Therefore, we next sought to determine whether SHK influences Nrf2 in hepatic L-02 cells. The results showed that SHK could both dose-dependently (Figure 4A) and time-dependently (Figure 4B) enhance the expression of Nrf2 in APAP-treated cells at the protein level. However, Figure 4C showed SHK had little effect on the Nrf2 mRNA level. Figure 4D–F revealed that SHK stimulated the mRNA expression of Nrf2-targeted genes including heme oxygenase (*HO1*), *GCLC* and *GCLM*, respectively. 

To reveal the significance of SHK-induced Nrf2 in APAP hepatic toxicity, we used brusatol, identified as an Nrf2 inhibitor [13,14], to inhibit Nrf2 transcriptional activity. The findings indicated that brusatol (160 nM) could efficiently reverse the effect of SHK on APAP-induced viability reduction (Figure 4G), GSH depletion (Figure 4H) and ROS accumulation (Figure 4I) in L-02 cells. These results strongly suggest that SHK ameliorates APAP hepatotoxicity via up-regulation of Nrf2 expression and resultant reduced oxidative stress. 

### 2.4. SHK Induces Nrf2 Expression Probably via PI3K/Akt/GSK3β Pathway in L-02 Cells

**Figure 5:**
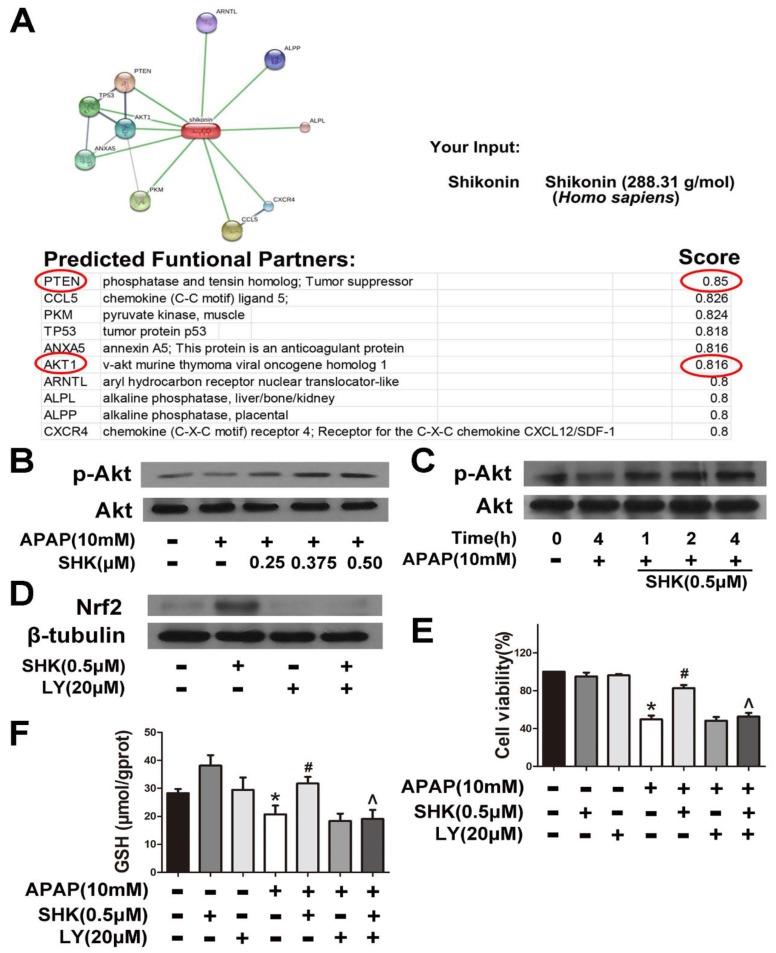
Akt phosphorylation is involved in SHK-mediated protective effects. (**A**) Protein and small molecule/chemical interaction analysis of SHK using STITCH (version 5.0). (**B**,**C**) L-02 cells were incubated with SHK for the indicated durations, and then the levels of Akt phosphorylation were evaluated by Western blotting. (**D**) L-02 cells were pretreated with LY294002 (20 μM) for 1 h, followed by treatment with SHK and LY294002 for 8 h, and then the Nrf2 protein levels were evaluated by Western blotting. (**E**,**F**) Cells were pretreated with LY294002 (20 μM) for 1 h, followed by treatment with SHK and LY294002 for 1 h, and then the cells were exposed to APAP for 24 h, then cell viability and GSH depletion was assessed. All of the data shown represent the average from three independent experiments. * *p* < 0.05 versus the control group; # *p* < 0.05 versus the APAP group; ^ *p* < 0.05 versus the SHK plus APAP group.

To determine the mechanism of SHK-induced Nrf2 expression, we used the online database STITCH to investigate the interactions between SHK and potential signaling pathways. As shown in Figure 5A, PTEN and its downstream Akt were predicted as functional partners of SHK. PI3K/Akt signaling is negatively regulated by PTENs [15] and, therefore, we proposed that SHK up-regulated Nrf2 expression via PI3K/Akt pathway. As shown in Figure 5B,C, SHK both dose-dependently and time-dependently enhanced Akt activity in APAP-treated cells as the level of p-Akt was elevated. Pre-incubation of cells with LY294002 (a PI3K inhibitor, 20 μM) for 1 h prevented the SHK (0.5 μM)-induced increase in Nrf2 expression, cytoprotection against APAP and GSH reversal under APAP exposure (Figure 5D–F). 

**Figure 6:**
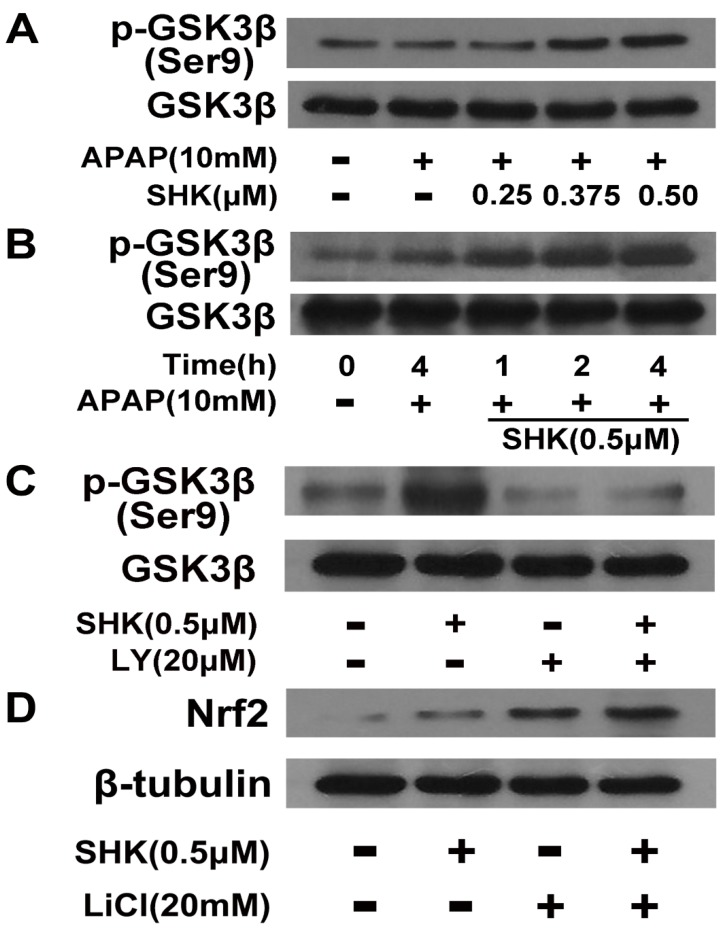
Effect of SHK on Akt-induced GSK3β phosphorylation and Nrf2 upregulation. (**A**,**B**) L-02 cells were incubated with SHK for the indicated durations, and then the levels of GSK3β phosphorylation were evaluated by Western blotting. (**C**) L-02 cells were pretreated with LY294002 (20 μM) for 1 h, followed by treatment with SHK for 4 h, and GSK3β protein and phosphorylated GSK3β levels were evaluated by Western blotting. (**D**) L-02 cells were pretreated with lithium chloride (20 mM) for 4 h, followed by treatment with SHK for 4 h, and GSK3β protein and phosphorylated GSK3β levels were evaluated by western blotting.

The results that SHK enhanced Nrf2 expression at the protein level while not at the mRNA level (Figure 4A–C) strongly suggest that SHK interfered Nrf2 stability. Nrf2 degradation is regulated by Keap1 and glycogen synthase kinase-3β (GSK-3β) [16], and GSK-3β is negatively regulated by PI3K/Akt axis via phosphorylation at Serine 9 (Ser 9) [17]. Therefore, we further determined whether SHK interferes Nrf2 stability via PI3K/Akt/GSK-3β pathway. As shown in Figure 6A,B, SHK increased GSK3β phosphorylation at Ser 9 in APAP-treated cells both dose- and time-dependently. In addition, treatment with LY294002 (20 μM) prevented the SHK-induced increases in GSK3β phosphorylation (Figure 6C). Figure 6D showed that lithium chloride (a classical GSK3β inhibitor, LiCl, 20 mM) could produce the similar effect on Nrf2 expression as SHK, and when GSK3β activity was blocked by LiCl, SHK treatment exerted not further increase in the Nrf2 protein level. Therefore, the above data clearly suggest that SHK regulates Nrf2 in a PI3K/Akt/GSK-3β-dependent manner. 

### 2.5. SHK Protects against APAP-Induced Hepatotoxicity via Akt/GSK3β/Nrf2 In Vivo

**Figure 7:**
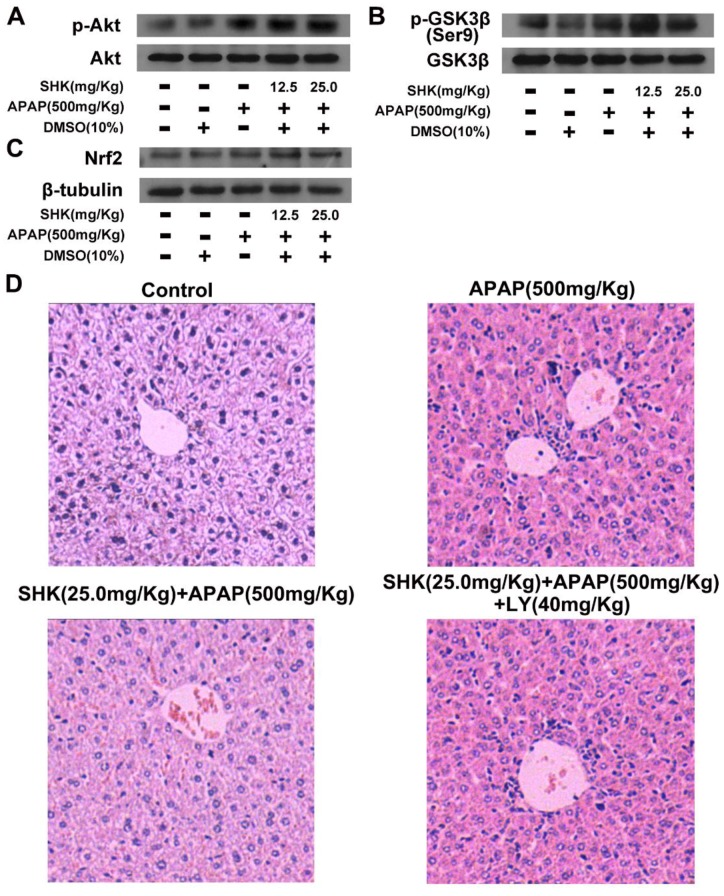
SHK protects APAP-induced hepatotoxicity via Akt/GSK3β/Nrf2 in mice. (**A**–**C**) Effects of SHK on phosphorylation of Akt and GSK3β, as well as Nrf2 protein expression analyzed by Western blotting. (**D**) LY294002 (40 mg/kg, i.p., 2 h before SHK administration), and then mice were administered SHK i.p. for 1 h. Then, the mice were injected with APAP for 9 h. Representative images of H&E staining of liver tissues.

We further examined the effects of SHK on the activities of Akt and GSK3β, as well as Nrf2 expression in liver tissues of APAP-treated mice by Western blotting assay. Similar with the in vitro findings, as shown in Figure 7A–C, SHK (12.5 and 25.0 mg/kg) administration enhanced the phosphorylation levels of Akt and GSK3β, as well as Nrf2 expression in liver tissues under APAP exposure, respectively. Furthermore, Figure 7D showed that APAP (500 mg/kg) elicited severe liver damage, and SHK (25.0 mg/kg) treatment substantially rescued such liver damage. Nevertheless, LY294002 (40 mg/Kg) could efficiently block the protective effect of SHK on APAP-induced liver damage.

## 3. Discussion

In the present study, we found that APAP overdose could induce an obvious toxicity in hepatocytes both in vitro and in vivo, accompanied by a significant ROS accumulation. Oxidative stress as GSH depletion and uncontrolled ROS is known to be the main pathogenesis of APAP-induced liver toxicity, inhibiting hepatocyte defense mechanisms [18]. 

Nrf2 controls cellular antioxidant responses through regulating the expression of enzymatic antioxidant systems (GPX, GR, PRX, and TRXR) to maintain redox homeostasis [19], and has been widely accepted to be associated with the process of APAP hepatotoxicity [20]. Interestingly, SHK has the potency in activating Nrf2 in multiple lines of normal cells. In this study, we found that SHK treatment could efficiently compromise APAP hepatic toxicity both in vitro and in vivo. SHK upregulated Nrf2 expression in hepatocytes, and inhibition of Nrf2 reversed the SHK effect on APAP-induced GSH depletion, ROS accumulation and hepatotoxicity. These data strongly support the idea that SHK protects APAP-induced liver toxicity via Nrf2 upregulation. 

This study found that SHK enhanced Nrf2 expression at the protein level while not at the mRNA level, strongly suggesting that SHK interfered with Nrf2 stability in hepatocytes. Next, we found that SHK stimulated PI3K/Akt pathway in APAP-treated cells and inhibition of PI3K/Akt signaling abrogated SHK-induced increase in Nrf2 expression under APAP exposure. These results revealed that SHK enhanced Nrf2 expression via the PI3K/Akt pathway. Nrf2 can be repressed by β-transducin repeat-containing protein (β-TrCP), present in the Skp1–cullin-1–F-box protein (SCF) ubiquitin ligase complex SCF/β-TrCP which is enhanced by phosphorylation of the transcription factor by GSK-3 [21,22]. GSK-3β is negatively regulated by PI3K/Akt axis via phosphorylation at Serine 9 (Ser 9) [17]. Therefore, we assumed that SHK might promote Nrf2 expression via the PI3K/Akt/GSK-3β pathway in hepatocytes. Our results indicated that SHK enhanced GSK-3β phosphorylation at Ser 9 in APAP-treated hepatocytes through PI3K/Akt activation. Furthermore, the data showed that SHK enhanced Nrf2 level dependent on GSK3β inhibition. These data together strongly suggest that SHK regulates Nrf2 stability in a PI3K/Akt/GSK-3β-dependent manner. 

The present study constructed the model of APAP overdose in mice to assess the protective effect of SHK against APAP hepatotoxicity in vivo. The results demonstrated that SHK attenuated APAP-induced liver toxicity in mice, as indicated by reduction in the serum ALT and AST levels and hepatocyte necrosis. GSH is a powerful antioxidant, cofactor, and coenzyme, closely involved in the clearance of ROS [23]. Our result revealed that SHK could improve GSH production and reduce the production of ROS. Furthermore, SHK decreased the MPO level, which is usually indicative of the extent of liver inflammation [24]. These results confirm that SHK has protective effects against APAP-induced oxidative stress and liver injury, consistent with our in vitro findings. To elucidate whether the liver protection of SHK is related to Akt/GSK3β/Nrf2 pathway in vivo, we tested the expression of associated proteins in mice liver. The results show SHK increased the levels of p-Akt, p-GSK3β and Nrf2 in the liver tissues, establishing the close correlation of liver protection of SHK against APAP and Akt/GSK3β/Nrf2 pathway in mice.

Our work has some limitations. DMSO is a very popular solvent in molecular biology, however, it is a potent ROS scavenger, especially, a scavenger of hydroxyl radicals [25]. Although we set up the solvent (DMSO) control group throughout the work, we cannot necessarily exclude the potential interactions between DMSO and shikonin. Therefore, for one hand, we should select superior solvent for individual experiment and certain tested drug used, and for another hand, we should be more rigorous to determine the possible interaction of solvent and tested drugs in future work. Moreover, though 2houghdichlorofluorescin diacetate (DCFH-DA) assay for intracellular detection of ROS is very popular, however, the deacetylation of DCFH-DA, even by esterases, can produce H_2_O_2_, thus acting as a redox cycler and may making detection of ROS somewhat complicated [26,27]. 

## 4. Materials and Methods 

### 4.1. Reagents and Chemicals

SHK, dimethyl sulfoxide (DMSO), LY294002, Lithium chloride (LiCl, a conventional GSK3β inhibitor), 2′,7′-Dichlorofluorescin diacetate (DCFH-DA) and 3-(4,5-dimethylthiazol-2-y1)-2,5-diphenyltetrazolium bromide (MTT) were obtained from Sigma-Aldrich (St. Louis, MO, USA). Brusatol was purchased from Tauto biotech (Shanghai, China). Antibodies against Nrf2 and β-tubulin were from Abcam (Cambridge, MA, USA). Antibodies against p-Akt, Akt, p-GSK3β Ser 9, and GSK3β were purchased from Cell Signaling (Boston, MA, USA). Alanine aminotransferase (ALT), aspartate aminotransferase (AST), glutathione (GSH), myeloperoxidase (MPO) test kits were acquired from Nanjing JianCheng Bioengineering Institute (Nanjing, China). APAP was purchased from MedChem Express (Monmouth, NJ, USA). 

### 4.2. Cell Culture and MTT Analyses

The L-02 cell line was obtained from Cell Bank, Type Culture Collection of Chinese Academy of Sciences, Shanghai. Cells were cultured in DMEM, 10% fetal bovine serum (FBS) at 37 °C with 95% air and 5% CO_2_. The cells (2 × 10^5^ cells/well) were treated with different concentrations of SHK (0.25, 0.375 and 0.50 μM) and APAP (10 mM) in the presence or absence of various inhibitors for 24 h. Then, an MTT solution (5 mg/mL) was added to the cells, and cultivated for 4 h. Finally, the MTT solution was abandoned, DMSO was added to each well, and absorbance was measured at 570 nm.

### 4.3. Detection of Intracellular Glutathione (GSH)

L-02 cells (2 × 10^5^ cells/well) were grown for 24 h, followed by treatment with SHK (0.25, 0.375 and 0.50 μM) for 4 h and exposure to APAP (10 mM) for 8 h. Then, GAH was assayed by a GSH test kit (Nanjing JianCheng Bioengineering Institute, Nanjing, China) according to the manufacturer’s instructions. 

### 4.4. Measurement of Cellular and Liver Reactive Oxygen Species (ROS)

The intracellular ROS was detected using a fluorescent probe DCFH-DA. After APAP treatment for 8 h, L-02 cells were washed with cold PBS and suspended in PBS at 5 × 10^5^ cells/mL. Then, the cells were mixed with DCFH-DA (5 μM) for 40 min at 37 °C in the darkness. The liver ROS was measured according to a previous report [28]. Briefly, cold liver homogenates were centrifuged (10,000× *g*, 15 min, 4 °C). The supernatants processed with 10 μM DCFH-DA in the dark for 1 h were transferred to a black wall with clear bottom 96-well plate. The relative fluorescence intensity was measured using a fluorescence spectrophotometer (HITACHI, 650-60, Tokyo, Japan) with the excitation wavelength of 485 nm and the emission wavelength of 530 nm.

### 4.5. Animals and Experimental Design

C57BL/6 mice (male, 6–8 weeks old, weighing approximately 18 to 22 g each) were purchased from Guangdong Medical Laboratory Animal Center (Guangzhou, China). Animal handling and procedures were approved by the Guangdong Pharmaceutical University Health Science Center Institutional Animal Care and Use Committee. All animal experiments complied with the National Institutes of Health guide for the care and use of laboratory animals (NIH Publications No. 8023, revised 1978).

Liver injury was induced by APAP (500 mg/Kg) dissolved in physiological saline. Blood samples were collected from retro-orbital venous plexus at 9 h after the injection of APAP. Then mice were dissected and liver tissues were removed immediately for subsequent assay. Mice were randomly divided into six groups (n = 5 for each group): (1) control group; (2) APAP group; (3) DMSO (10%) as a solvent control group; (4, 5) SHK (12.5 and 25.0 mg/Kg) + APAP group. SHK was dissolved in DMSO and injected intraperitoneally (i.p.) to treated mouse at 1 h before the injection of APAP; (6) SHK (25.0 mg/Kg) + APAP + LY294002 (40 mg/kg) group. LY294002 was administrated 2 h before SHK injection by i.p. 

### 4.6. Biochemical and Histological Analyses

The mice were sacrificed at 9 h after APAP treatment. Blood was collected and centrifuged to obtain serum. The serum ALT and AST levels were detected using an assay kit according to the manufacturer’s instructions. The mouse liver tissues were homogenized in cold metaphosphoric acid (5%) buffer, and centrifuged at 10,000× *g* for 10 min, then the supernatant was transferred to new tubes. The GSH and MPO were analyzed according to the manufacturer’s instructions. Fresh liver tissues were fixed in 4% paraformaldehyde, sectioned, embedded in paraffin and cut into 2 μm sections, and subsequently stained with H&E for pathological analysis.

### 4.7. Western-Blot Analysis

Protein samples were detached by sodium dodecyl sulfate polyacrylamide gel electrophoresis (SDS-PAGE) gel electrophoresis and then electrophoretically transferred to polyvinylidene difluoride (PVDF) membrane. The membranes were probed with primary antibodies and horseradish peroxidase-conjugated secondary antibodies. Proteins in the membranes were visualized by enhanced chemiluminescence kits. 

### 4.8. Real-Time Polymerase Chain Reaction (PCR) Analysis

Total RNA was extracted from L-02 cells using Trizol Reagent (Invitrogen), and then complementary DNA (cDNA) was synthesized using ReverTra Ace reverse transcriptase (TOYOBO, Osaka, Japan, FSQ-301) according to the manufacturer’s protocol. The process was performed with the SYBR Green Realtime PCR Master Mix (TOYOBO, Osaka, Japan, QPK-201) on an iCycler (Bio-Rad, Hercules, CA, USA) following the manufacturer’s protocol. The primers were shown in supplementary Table S1. The gene expression levels for each amplication were calculated using the ΔΔCT method [29] and normalized against GAPDH mRNA.

### 4.9. Statistical Analysis

Data were presented as means ± S.D. and analyzed with the unpaired Student’s test by using GraphPad 5 Software. *p* value of <0.05 was considered statistically significant.

## 5. Conclusions

The present study indicates that SHK protects APAP-induced liver injury via inhibition of oxidative stress through AKT/GSK3β pathway-dependent Nrf2 upregulation in hepatocytes. Hence, SHK may be a promising candidate for treatment of APAP hepatotoxicity. 

## Supplementary Materials

The supplementary materials are available online.

## Abbreviations

DILI	Drug-induced liver injury
APAP	Acetaminophen
SHK	Shikonin
ALT/AST	Alanine/aspartate aminotransferase
GSH	Glutathione
ROS	Reactive oxygen species
MPO	Myeloperoxidase
GSK3β	Glycogen synthase kinase-3β
Akt	Protein Kinase B
Nrf2	Nuclear factor erythroid 2-related factor 2
HO-1	Heme oxygenase 1
DCFH-DA	2′,7′-Dichlorofluorescin diacetate
LiCl	Lithium chloride
MTT	3-(4,5-dimethylthiazol-2-y1)-2,5-diphenyltetrazolium bromide
