# Correction: Li et al. Shikonin Attenuates Acetaminophen-Induced Hepatotoxicity by Upregulation of Nrf2 through Akt/GSK3β Signaling. *Molecules* 2019, *24*, 110

**DOI:** 10.3390/molecules28145377

**Published:** 2023-07-13

**Authors:** Huachao Li, Yueming Chen, Jiahao Zhang, Xiangcui Chen, Zheng Li, Bing Liu, Luyong Zhang

**Affiliations:** 1 Department of Pharmacology, School of Pharmacy, Guangdong Pharmaceutical University, Guangzhou 510006, China; rehclee27@163.com (H.L.); 18766979213@163.com (X.C.); 2 Guangzhou Key Laboratory of Construction and Application of New Drug Screening Model Systems, Guangdong Pharmaceutical University, Guangzhou 510006, China; 3 Key Laboratory of New Drug Discovery and Evaluation of Ordinary Universities of Guangdong Province, Guangdong Pharmaceutical University, Guangzhou 510006, China; 4 The Center for Drug Research and Development, Guangdong Pharmaceutical University, Guangzhou 510006, China

The authors wish to make the following corrections to this paper [1]:

Figure 6 contains some errors, and should be replaced with the correct one. During the several rounds of figure assembly, the following mistakes were unintentionally made by the authors: the GSK3β band of Figure 6C and the β-tubulin band of Figure 6D are introduced from the same original β-tubulin band in Figure 5D. In addition, the Nrf2 band of Figure 4A was mistakenly selected, as it was the short exposure of the Nrf2 band in Figure 4B. The authors have provided correct versions of Figure 4A and Figure 6.

The authors state that the scientific conclusions are unaffected. This correction was approved by the Academic Editor. The original publication has also been updated.

## Figures and Tables

**Figure 4:**
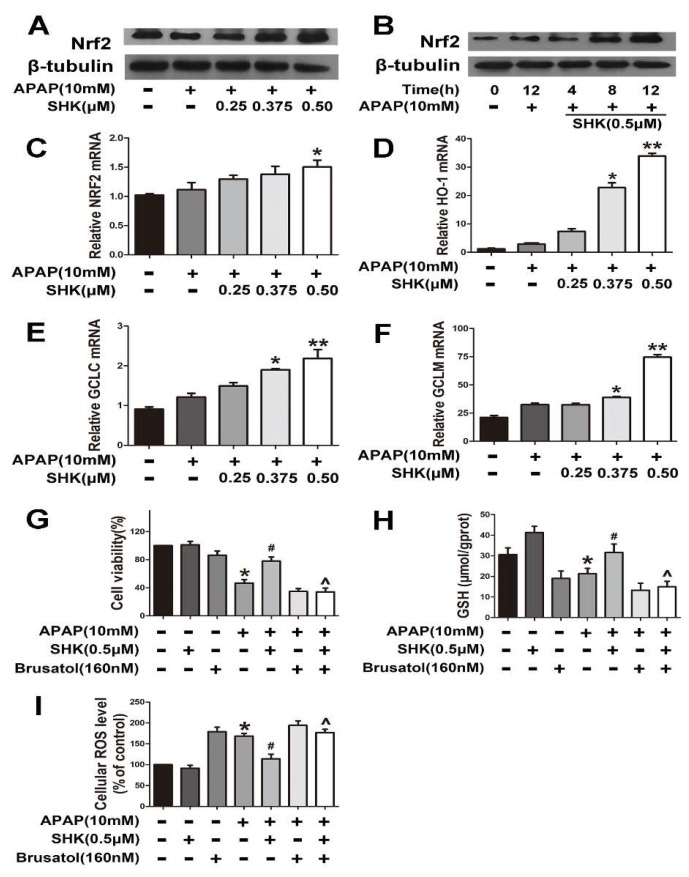
The effects of SHK are dependent upon the Nrf2 in L-02 cells. (**A**,**B**) L-02 cells were treated with SHK for the indicated durations, and then the protein levels were examined via Western blot analysis. (**C**–**F**) L-02 cells were treated with increasing concentrations of SHK for 1 h followed by 4 h APAP treatment, and then the mRNA expression of Nrf2, *HO-1*, *GCLc* and *GCLm* was detected by real-time polymerase chain reaction (PCR). (**G**–**I**) Cells were pretreated with brusatol (160 nM) for 2 h, followed by treatment with SHK for 1 h, and then the cells were exposed to APAP for 24 h. Cell viability, GSH and ROS level were assessed. All of the data shown represent the average from three independent experiments. * *p* < 0.05 versus the control group; ** *p* < 0.01 versus the control group; # *p* < 0.05 versus the APAP group; ^ *p* < 0.05 versus the SHK plus APAP group.

**Figure 6:**
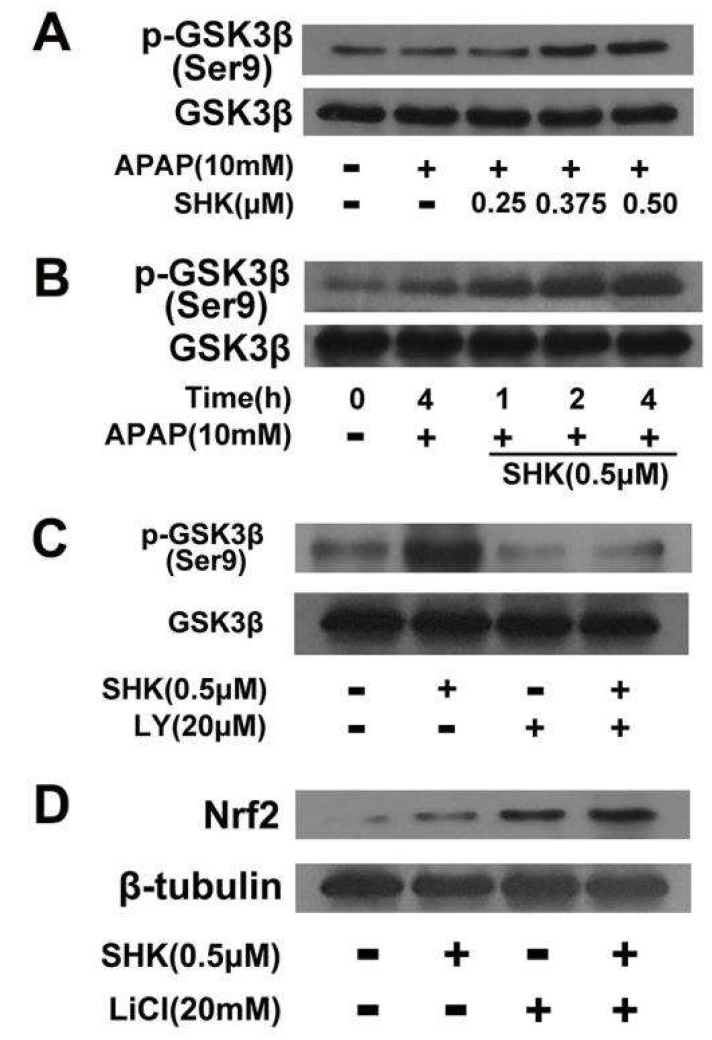
The effect of SHK on Akt-induced GSK3β phosphorylation and Nrf2 upregulation. (**A**,**B**) L-02 cells were incubated with SHK for the indicated durations, and then the levels of GSK3β phosphorylation were evaluated via Western blotting. (**C**) L-02 cells were pretreated with LY294002 (20 μM) for 1 h followed by treatment with SHK for 4 h, and GSK3β protein and phosphorylated GSK3β levels were evaluated via Western blotting. (**D**) L-02 cells were pretreated with lithium chloride (20 mM) for 4 h, followed by treatment with SHK for 4 h, and GSK3β protein and phosphorylated GSK3β levels were evaluated via Western blotting.
