# Supplementary material for: Shikonin Attenuates Acetaminophen-Induced Hepatotoxicity by Upregulation of Nrf2 through Akt/GSK3β Signaling
Source: Molecules. 2018 Dec 29;24(1):110. doi: 10.3390/molecules24010110 (PMC6337349; doi:10.3390/molecules24010110)
Supplement: Supplementary file 1 [file molecules-24-00110-s001.pdf]

Shikonin attenuates acetaminophen-induced hepatotoxicity by upregulation of Nrf2 through Akt/GSK3β signaling

Huachao Li[1,2,3#], Yueming Chen[1,2,3#], Jiahao Zhang[1,2,3], Xiangcui Chen[1,2,3], Zheng Li[2,3], Bing Liu[1,2,3*], Luyong Zhang[2,3,4*]

1 Department of Pharmacology, School of Pharmacy, Guangdong Pharmaceutical University, Guangzhou 510006, China; 2860351264@qq.com (H.L.); 516372987@qq.com (Y.C.); avlsunny@126.com (J.Z.); 1149290924@qq.com (X.C.).

2 Guangzhou key laboratory of construction and application of new drug screening model systems, Guangdong Pharmaceutical University, Guangzhou 510006, China; li.zheng.sky@163.com (Z.L.).

3 Key Laboratory of New Drug Discovery and Evaluation of ordinary universities of Guangdong province, Guangdong Pharmaceutical University, Guangzhou 510006, China

4 The Center for Drug Research and Development, Guangdong Pharmaceutical University, Guangzhou 510006, China*

Correspondence: Luyong Zhang, Email: lyonzhang@163.com. Tel: 86-20-39352100, Fax: 86-20-39352100; Bing Liu, Email: liubing52000@163.com. Tel: 86-20-39352128.

Table S1 Oligo nucleotide sequence of PCR primers

Primers for RT-PCR	Sequence-forward(5' -3')	Sequence-backward(5' -3')
Nrf2	GACGTGTGGCGGCTGAGC	GCACCGCGTCCGAACTAGAAG
HO-1	GGTGCTCGTACTGCTACTGTCATG	GCCACGAACCTCATCTCTTCCAC
GCLC	GGAGGAGGAGGAGGAGGAGGAG	GGCGTGGTAGATGTGCAGGAAC
GCLM	CGCAGTGGCTCACACCTGTAATC	GTTCAAGCGATTCTCCTGCCTCAG
